# Current Overview of Allergens of Plant Pathogenesis Related Protein Families

**DOI:** 10.1155/2014/543195

**Published:** 2014-02-16

**Authors:** Mau Sinha, Rashmi Prabha Singh, Gajraj Singh Kushwaha, Naseer Iqbal, Avinash Singh, Sanket Kaushik, Punit Kaur, Sujata Sharma, Tej P. Singh

**Affiliations:** Department of Biophysics, All India Institute of Medical Sciences, Ansari Nagar, New Delhi 110029, India

## Abstract

Pathogenesis related (PR) proteins are one of the major sources of plant derived allergens. These proteins are induced by the plants as a defense response system in stress conditions like microbial and insect infections, wounding, exposure to harsh chemicals, and atmospheric conditions. However, some plant tissues that are more exposed to environmental conditions like UV irradiation and insect or fungal attacks express these proteins constitutively. These proteins are mostly resistant to proteases and most of them show considerable stability at low pH. Many of these plant pathogenesis related proteins are found to act as food allergens, latex allergens, and pollen allergens. Proteins having similar amino acid sequences among the members of PR proteins may be responsible for cross-reactivity among allergens from diverse plants. This review analyzes the different pathogenesis related protein families that have been reported as allergens. Proteins of these families have been characterized in regard to their biological functions, amino acid sequence, and cross-reactivity. The three-dimensional structures of some of these allergens have also been evaluated to elucidate the antigenic determinants of these molecules and to explain the cross-reactivity among the various allergens.

## 1. Introduction

Plants are one of the major sources of allergens which elicit allergenic response by immunoglobulin E (IgE) mediated allergies [1, 2]. These allergens may diffuse into the body from the upper respiratory tract or enter the body through intake of vast range of plant food or may cause external skin irritations [3–5]. Allergens present primarily in pollens, spores, and other plant associated products are responsible for symptoms like rhinoconjunctivitis, asthma, edema, urticarial, and anaphylaxis [6–8]. Allergens ingested as food result in responses like pruritus and swelling of lips, tongue, and soft palate, often accompanied by mild laryngeal symptoms as a sensation of tightness, itching, cough, gastrointestinal symptoms, rhinitis, asthma, cutaneous reactions, and more severe systemic anaphylaxis [9–12]. Some plant derived allergens result in contact dermatitis mostly in skin like itchy fingers, skin irritations, and so forth [13, 14]. The most widespread groups of plant allergens that are reported belong to the seed storage proteins, structural proteins, and pathogenesis related (PR) proteins [15–17].

Allergens are assigned names based on their accepted taxonomic nomenclature with the first three letters designating their genus and followed by the first letter of the species and an Arabic number denoting the order of the identification. The allergenicity of the protein is mostly dependent on the structural motifs present in the allergens which act as the allergenic determinants or the epitopes that are responsible for binding to B and T cells. B cell epitopes are mostly discontinuous motifs forming conformational epitopes whereas T cell epitopes are linear and continuous [18, 19]. It has been found that allergens from diverse species possess similar structural motifs that can be identified by the antibodies resulting in IgE cross-reactivity.

## 2. Plant Pathogenesis Related Proteins

Plant pathogenesis related (PR) proteins are generally induced by various types of pathogens such as viruses, bacteria, and fungi [20–22]. Some of these proteins are also expressed in response to some chemicals that act in a similar way as pathogen infection [23, 24]. However, some of the PR proteins are constitutively expressed in some organs or during certain developmental stages [25, 26]. They are regarded as PR-like proteins because of their sequence homology. However, some PR-like proteins are found to be strongly induced by infections and hence they are also designated mostly as PR proteins [27].

PRs were first identified from tobacco leaves (*Nicotiana tabacum*) infected with tobacco mosaic virus and later have been detected in numerous plants of different species [28]. They exhibit distinct biochemical characteristics which are necessary when the plant is under pathogenic infections or any unwanted stress. They are generally low-molecular weight proteins in the range 6 to 43 kDa, stable at low pH (<3), and are protease resistant which helps them survive in the harsh conditions like the vacuolar compartment, cell wall, or intercellular spaces [20]. Depending on their isoelectric points, PR proteins are either acidic or basic and are also found to be either vacuolar or apoplastic [29]. The acidic forms of PR proteins are mostly secreted to the extracellular space and the basic forms are transported to the vacuole by a signal located at the C-terminus [30]. However, such localization cannot be generalized for all PR proteins except in the case of certain tobacco PR family. Currently, PRs are found to be localized in almost all plant organs including leaves, stems, roots, and flowers, though maximum abundance of these proteins is found in the leaves [29].

Usually, upregulation of gene expression during pathogen attack takes place by various signaling molecules like salicylic acid [23, 31, 32] and reactive oxygen species [33] which mediate the expression of acidic PR genes. Induction of basic PR genes is mediated by gaseous phytohormone ethylene and methyl jasmonate [24]. Apart from various environmental factors that trigger the synthesis of these PR proteins, their expressions are also dependent on certain internal developmental stimuli of the plant.

Originally 5 groups of PR proteins have been identified [34] but gradually with the increasing identification of new PR proteins, presently, 17 families of PR proteins are recognized based on their amino acid sequence similarities, enzymatic activities, or other biological properties and numbered in the order in which they were discovered (Table 1) [29, 35, 36]. In spite of their common name, these proteins display a great diversity in species specificity and in the mechanism of action and do not share any structural relationship among themselves.

PR proteins exhibit multiple functions within the plant. Most PRs exhibit antifungal activity [37, 38] though antibacterial, insecticidal, nematicidal, and antiviral activity of some of the PR proteins have also been reported [39, 40]. Some of the PR proteins have enzymatic functions like *β*-1,3-glucanase [41] or chitinase activities [42]; some including defensins [43] and lipid transfer proteins [44] have membrane permeabilizing effect. PR proteins thus have crucial function in disease resistance, seed germination, and plants facilitation to adapt to the environmental stress.

Apart from PR proteins, plants under pathogen attack produce other families of proteins having defensive action like PR proteins. Two *α*-amylases are found to be induced in tobacco upon TMV infection [45]. Polygalacturonase inhibitor proteins (PGIPs) are produced by pathogen infection and stress-related signals in plants that can inhibit fungal endopolygalacturonases [46]. Others include cell wall hydroxyproline-rich glycoproteins [47] and lipoxygenases [48]. Certain plant storage proteins, like 2S-albumins, lectins, vicilins, glycine-rich proteins, and so forth, accumulate in storage vacuoles inside plant cells and perform essential roles as antimicrobial agents in response to pathogen attack [49, 50]. Ribosome-inactivating proteins (RIPs), cysteine-rich peptides, and so forth are also expressed by plants as antimicrobial molecules but are not induced by any pathogen attack [51, 52].

## 3. Plant PR Proteins and Allergenicity

In the recent years, a variety of PR proteins and their homologues causing allergenicity in humans have been isolated and characterized [53–56]. The size, stability, and resistance to proteases along with hydrolytic and membrane-permeabilizing ability in some make these proteins excellent candidates to elicit allergenic response [53]. Moreover, PR proteins are mostly associated with high degree of cross-reactivity because of structural similarity among some of the major proteins. Many patients allergic to one form of allergen like pollen also display allergic symptoms after ingesting some other allergens like certain fresh fruit, vegetable, or nut [57–59]. Thus, IgE antibodies originally produced in response to a particular allergen sensitization recognize comparable epitopes which are present on the surface of other plant proteins. Hence, reexposure to homologous plant allergens induces an allergic reaction in already sensitized individuals. Some of the common allergic syndromes like pollen-related food syndrome, latex-fruit syndrome, or the birch-mugwort-celery-spice syndrome are associated among the different PR proteins [60–62].

It has also been observed that plants growing under different conditions have varied levels of expression of the allergenic PR proteins and their homologues [63]. The expression of some of these classes of allergens also shows alterations due to environmental pollutants [55]. Based on sequence characteristics, a number of allergens classified as PR proteins are recognized from PR families 1, 2, 3, 4, 5, 8, 10, and 14 (Table 1) [29]. Thus, evaluating the structure and role of members of these different PR families in allergenicity will help to understand the allergenic cross-reactivity and will explain the differences in the frequency of sensitization and severity of allergenicity in sensitized individuals.

### 3.1. PR-1 Family Allergens

PR-1 proteins were first found to be expressed in tobacco in response to tobacco mosaic virus (TMV) infection having 14 to 17 kDa molecular weights [64]. Later, homologues of tobacco PR-1 proteins have been identified in barley, tomato, maize, rice, and so forth [65–68]. These widely distributed proteins of plant kingdom have antifungal activity at the micromolar level against a number of plant pathogenic fungi [66], but their mechanism of action is not known. No allergens were reported from PR-1 protein family till 2004. The first evidence of an allergen Cuc m 3 was reported from muskmelon which comprises many pollen allergens, thus delivering the involvement of this plant allergen family in food allergy [69]. Cuc m 3 shows more than 60% of sequence identity with PR-1 members from grape and cucumber.

### 3.2. PR-2 Family Allergens

PR-2 family of proteins are *β*-1,3-glucanases (glucan endo-1,3-*β*-glucosidases) which are monomeric enzymes having molecular weight around 20–23 kDa. These highly regulated enzymes catalyze the hydrolytic cleavage of *β*-1,3-glucans abundantly present in plant cell walls [70]. These enzymes function in response to pathogenic attack and are also involved in several physiological and developmental processes, for example, cell division [71], microsporogenesis [72], pollen germination [73], fertilization [74] and seed germination [75], and mobilisation of storage products in the endosperm of cereal grains [76]. These proteins were also induced in response to ozone and ultraviolet B light, mechanical injury, and freezing temperatures [77–79].

The PR-2 proteins are divided into three classes based on amino acid sequence identity, primary structure, cellular localization, and mode of expression [80]. The class I members with approximate size of 33 kDa are basic and localized in the cell vacuole and are found in tobacco, tomato, potato, and other plant species [81]. The class II and class III proteins are acidic proteins with average molecular weights around 34 to 36 kDa secreted into the extracellular space [82]. Antifungal activity has been observed only in class I *β*-glucanases. The proteins belonging to class I family have an additional C-terminal extension which is posttranslationally cleaved during intracellular transport and are likely to contain the vacuolar targeting signal [83].

**Figure 1:**
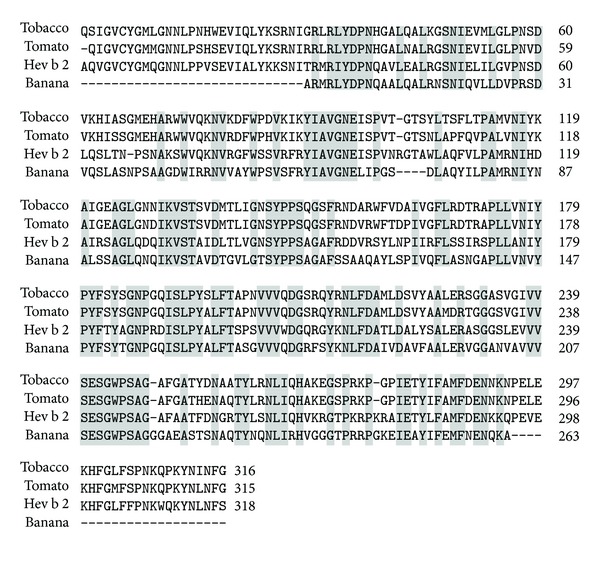
Multiple sequence alignment of *β*-1,3-glucanases from tobacco, tomato, banana, and latex (Hev b 2). The identical residues are highlighted in grey.

Some of the allergens having sequence similarity to *β*-1,3-glucanases have been identified. Latex from *Hevea brasiliensis* contains several allergenic proteins that are involved in allergenicity resulting in symptoms like mild contact urticaria to asthma and anaphylactic reactions that frequently occur during surgical or endoscopic procedures [84, 85]. One of them is Hev b 2, a *β*-1,3-glucanase enzyme that is recognized by IgEs of latex-allergic patients. This protein shares 62.9% and 64.7% sequence identities with tobacco and tomato *β*-1,3-glucanases (Figure 1). This 36 kDa protein is present in different isoforms and with variable glycosylation content [86, 87]. Both its peptidic and carbohydrate moieties are known to possess allergenic determinants [88, 89].

**Figure 2:**
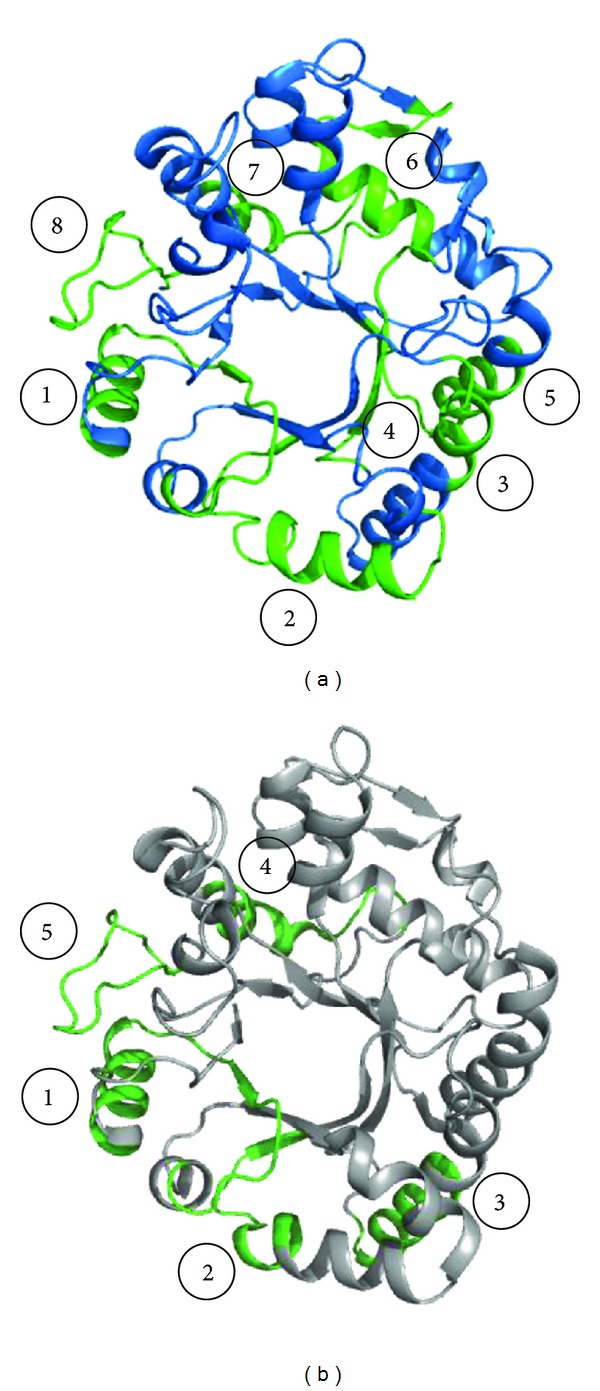
(a) Overall structure of Hev b 2 showing IgE binding epitopes (in green). (b) Overall structure of *β*-1,3-glucanase from banana showing IgE binding epitopes (in green).

The structure of Hev b 2 adopts a TIM-barrel, (*α*/*β*)_8_ fold (PDB code: 3EM5). Several IgE binding epitopes have been recognized along the entire amino acid sequence of the major latex allergen Hev b 2 [90] (Figure 2(a)). The amino acid residues residing in the IgE binding epitopic regions are found to be mostly exposed on the surface and the epitopes usually correspond to charged regions on the molecular surface of the protein.

*Hevea* latex allergy has been found to be associated with hypersensitivity to foods, especially avocado, banana, chestnut, fig, bell pepper, and kiwi, and is termed latex-fruit syndrome [91–93]. The reason for such cross-reactivity is that the proteins expressed in these fruits share very similar overall conformation and charge distribution to those of Hev b 2. Hev b 2 has about 60.8% sequence identity with banana *β*-1,3-glucanase, Mus a 5, the expression of which increases to a considerable amount during fruit ripening [94]. Five IgE binding epitopes similar to Hev b 2 have been identified in this protein (Figure 2(b)).

**Figure 3:**
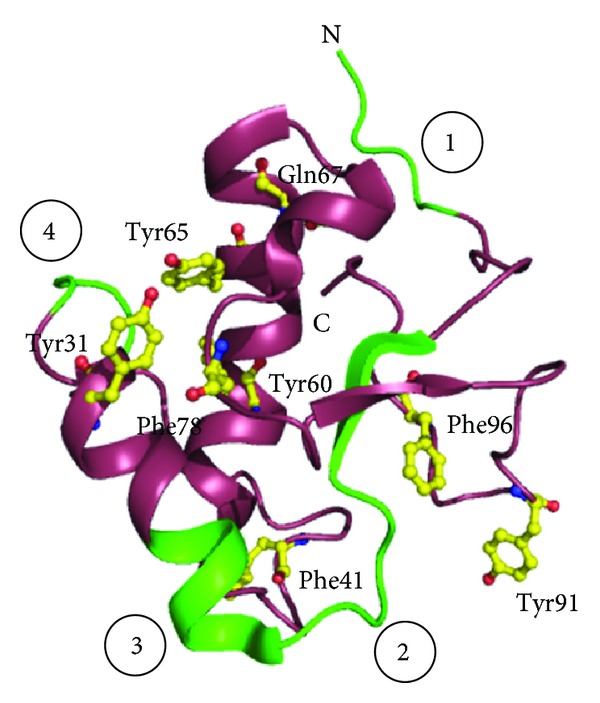
Structure of C-terminal domain of Ole e 9 from olive pollen. The four IgE binding epitopes (in green) marked from 1 to 4 are shown. The aromatic residues which form two distinct sugar binding faces are shown in yellow.

Another PR-2 protein, Ole e 9, has been characterized from olive pollen [95]. The protein is composed of two immunological independent domains: an N-terminal domain with *β*-1,3-glucanase activity and a C-terminal domain that binds 1,3-*β*-glucans. The overall structure of C-terminal domain of Ole e 9 has been found to comprise two parallel *α*-helices, a small antiparallel *β*-sheet with two short strands, and a 3–10 helix turn connected with each other by long coil segments (Figure 3). Two regions have been identified on the protein surface which are constituted of aromatic residues and have a possible role in sugar binding. Using epitope mapping four IgE epitopes have been characterized on the C-terminal domain of Ole e 9 which are mainly concentrated on the loops and few in secondary structural elements [96].

### 3.3. PR-3 Family Allergens

Among the seven different classes of chitinases, chitinases of classes I, II, and IV are grouped under PR-3 family proteins. Chitinases hydrolyze the glycosidic bonds in chitin, a component of the cell walls of fungi and exoskeletal elements of some animals [97]. Plant chitinases are monomeric proteins of 25–35 kDa molecular weights and are mostly endochitinases which break the chitin within the biopolymer. They produce 2–6 N-acetyl-glucosamine units and hydrolyze *β*-1,4-linkages between the N-acetylmuramic acid and the N-acetylglucosamine of lysozyme.

Some of the class I chitinases found in seed producing plants are basic proteins that are vacuolar and antifungal, whereas the acidic ones are extracellular with little antifungal activity. This class of chitinases contains a cysteine-rich 40-amino-acid domain at the N-terminus, the chitin binding hevein domain, a hypervariable domain (which is a proline-rich hinge region), and a catalytic domain [98]. The exact role of the hevein domain is not clear though it is required for chitin binding and for substrate affinity. It is speculated that the chitin-binding domain helps in increasing the efficiency of enzymatic cleavage of the polymer by attaching the catalytic domain onto the substrate. The intracellular localization depends on the presence of a C-terminal vacuolar targeting propeptide. A signal peptide is removed from the mature protein and a target sequence directing the protein to the vacuole is located at the C-terminus.

Class II chitinases having molecular weights of 27 to 28 kDa resemble class I proteins in terms of amino acid sequence, but they lack the N-terminal cysteine-rich hevein domain and the vacuolar target sequence. These enzymes are mainly acidic and extracellular and display 60–65% sequence similarities to class I chitinases. Some of these members induce antifungal activity in living plant cells rather than killing the invading fungus. Class IV proteins are similar to class I chitinases but are significantly smaller in size due to four major deletions [99].

**Figure 4:**
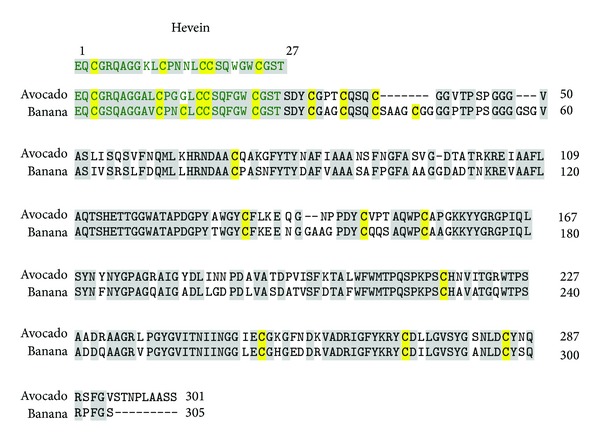
Sequence alignment of chitinase I from avocado (Pers a 1) and banana. The hevein-like domain (matching with sequence of hevein (1–27)) has also been marked in green. The identical residues and the cysteine residues are highlighted in grey and yellow, respectively.

Some of the major class I chitinase allergens of the PR-3 family have been identified from chestnut (Cas s 5) and avocado (Pers a 1) [100, 101]. Since these proteins are characterized by the presence of a conserved hevein-related structure, patients with previous exposure to latex prohevein or hevein are potential candidates for cross-reaction with these fruits and vegetables [102]. Pers a 1, a 32 kDa endochitinase, exhibits strong antifungal activity [101]. Class I chitinases are also recognized in banana (Mus a 2) with hevein-like domain having IgE binding properties. This protein shares about 74.0% amino acid sequence identity with Pers a 1 including the hevein-like domain present in both proteins (Figure 4). The stability of the proteins is maintained by the presence of cysteine residues. However, the three-dimensional structures of these proteins have not yet been reported [103].

### 3.4. PR-4 Family Allergens

**Figure 5:**
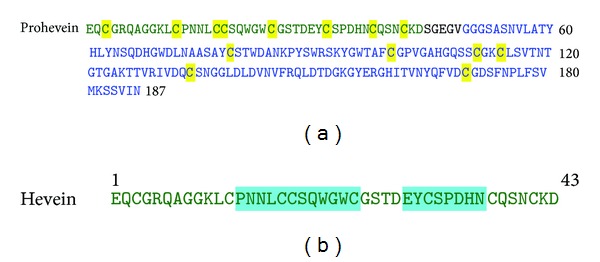
(a) Amino acid sequence of prohevein showing two allergenic domains obtained after posttranslational cleavage: Hev b 6.02 domain (green) and Hev b 6.03 domain (blue). 14 cysteine residues are highlighted in yellow. (b) Amino acid sequence of hevein showing the IgE binding epitopes highlighted in cyan.

**Figure 6:**
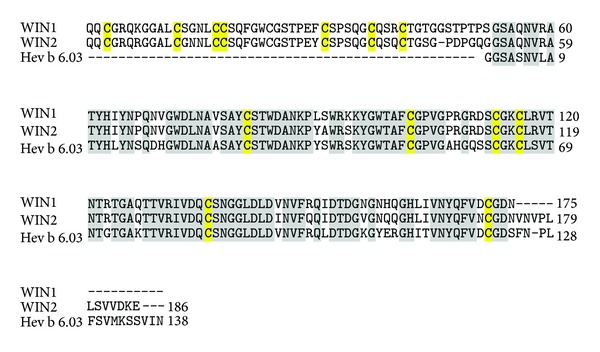
Multiple sequence alignment of Hev b 6.03 with wound inducible proteins WIN 1 and WIN 2. The identical residues are highlighted in grey and the cysteine residues in yellow.

PR-4 proteins are chitin-binding proteins, having molecular weights around 13 to 14.5 kDa. Some of the PR-4 proteins identified are tobacco protein CBP-20 and barley barwin [104, 105]. The common class I chitinases representing this family are prohevein and wound-inducible proteins. Prohevein, a cysteine-rich 20 kDa protein from *Hevea brasiliensis*, is designated as Hev b 6.01 and is one of the major IgE binding allergens in natural rubber latex allergy, especially common in health care workers [106]. It contains 14 cysteine residues that stabilize its tertiary conformation by forming multiple disulphide bridges. After posttranscriptional processing, prohevein generates the 4.7 kDa N-terminal Hev b 6.02 (hevein) and the 14 kDa C-terminal Hev b 6.03 (Figure 5(a)) both of which are allergenic. The former is involved in IgE binding and carries discontinuous B cell epitopes (Figure 5(b)), whereas Hev b 6.03 is responsible for proliferation response and contains human leucocyte antigen, HLA-DR4-binding motifs [107]. Hev b 6.03 shares more than 90% sequence similarity with wound-inducible proteins like potato stress proteins, WIN1 and WIN2 (Figure 6).

**Figure 7:**
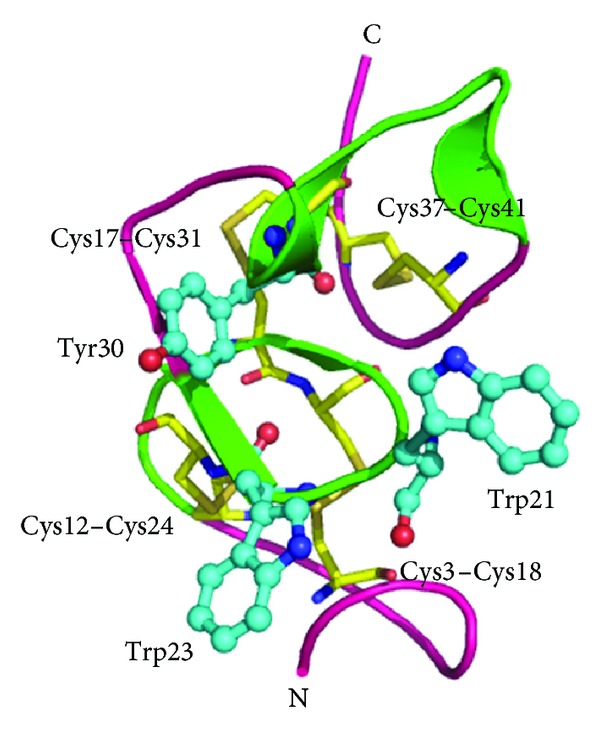
Overall structure of Hev b 6.02 (hevein) showing IgE binding epitopes (in green). Trp21, Trp23, and Tyr30 form an aromatic patch in the conformational epitope. The four disulphide linkages are shown in yellow.

Hevein has significant sequence similarities (about 71.7%) with chitin-binding proteins of PR-3 and PR-4 families [106] and is one of the reasons of latex allergic patients resulting in food allergies. Hevein is the major cross-reacting allergen with avocado in subjects with latex allergy [108, 109]. The crystal structure of hevein is folded into a series of loops all linked together by four disulfide bonds (Figure 7). An aromatic patch is formed in the central part of the protein by 2 tryptophan and 1 tyrosine residues encircled by 4 glutamate side chains forming a carbohydrate-combining site constituting a conformational epitope [110]. Current studies have shown that hevein is an ideal target for application in latex immunotherapy. Hypoallergenic variants of prohevein have been obtained by site directed mutagenesis in hevein domain which showed attenuated B cell reactivity but retained human T lymphocyte reactivity [111].

The class II chitinases belonging to PR-4 proteins have been identified from tobacco and tomato with sequence similarity to win proteins, yet lacking the chitin-binding domain [112]. Whole or wounded turnips treated with salicylic acid, ethephon, or water resulted in the expression of an 18.7 kDa protein exhibiting appreciable allergenicity that was recognized by IgE of natural rubber latex allergic patients [113].

### 3.5. PR-5 Family Allergens

PR-5 proteins have high amino acid homology to sweet tasting protein, thaumatin, from the South African berry bush *Thaumatococcus daniellii *[114] and are known as thaumatin-like proteins (TLPs) though none of these proteins have been reported to have a sweet taste. These proteins were first identified in tobacco leaf extracts when the plant was infected with tobacco mosaic virus [115]. Though TLPs have been mostly observed in leaves of young plants, they are detected in high levels upon biotic or abiotic stress. Osmotin and NP24 proteins from tobacco and tomato, respectively, are homologous to thaumatin and accumulate after osmotic stress [116, 117]. TLPs are also developmentally expressed to a significant amount in flower buds of turnip and overripe fruits of cherries [118, 119].

Based on their molecular weight, proteins of this class are grouped into two types: the first class having molecular weights ranging from 22 to 26 kDa and the other class having molecular weights around 16 kDa due to an internal deletion of 58 amino acids. Generally, they are acidic, basic, or neutral TLPs. Some of these proteins exert antifungal activity possibly by directly inserting them into the fungal membrane forming a transmembrane pore, eventually resulting in influx of water followed by osmotic rupture. Zeamatin, an antifungal 22 kDa protein that acts by causing membrane permeabilization has been reported, corn seeds [120]. Similar proteins with considerable sequence homology and similar antifungal action are also reported from tobacco, oats, sorghum, and wheat [121].

The allergens of this group are mainly pollen or food derived allergens. Several pollen allergens like Jun a 3 (mountain cedar) [63], Cry j 1 (Japanese cedar) [122], Cup a 3 (Arizona cypress) [123], and Jun v 3 (Eastern red cedar) [124] have been reported. Some of these allergens like Jun a 3 showing variability of expression may contribute to variable allergenicity in different lots of pollen. Four IgE binding epitopes have been predicted in the sequence of Jun a 3: Ala120 to Lys131, Val132 to Lys144, Asn152 to Lys165, and Asn169 to Lys179. Jun a 3 has been found to exhibit cross-reactivity with Cry j 1, from Japanese cedar (*Cryptomeria japonica*) [63]. Cup a 3 from Arizona cypress (*Cupressus arizonica*) is homologous to Jun a 3 and shows increased allergenicity of pollen from industrialized areas [123].

Allergens of this group belonging to food allergens include Pru av 2 from cherries [125], Cap a 1 from bell pepper [62, 126], Mal d 2 from apple [127], Act d 2 from kiwi [128], and Pru p 2 from peach [129]. Pru av 2, the major allergen in cherries, is one of the main causes of oral allergy syndrome and shares considerable sequence identity with Jun a 3. Thaumatin-like protein, associated with baker's respiratory allergy, has been also identified in wheat (*Triticum aestivum*) [130]. A 24 kDa protein from grapes, homologous to Pru av 2, was reported as a minor allergen [131]. Cap a 1 identified from bell pepper shows an IgE-mediated contact allergy in patients with the mugwort-birch-celery-spice syndrome [62].

**Figure 8:**
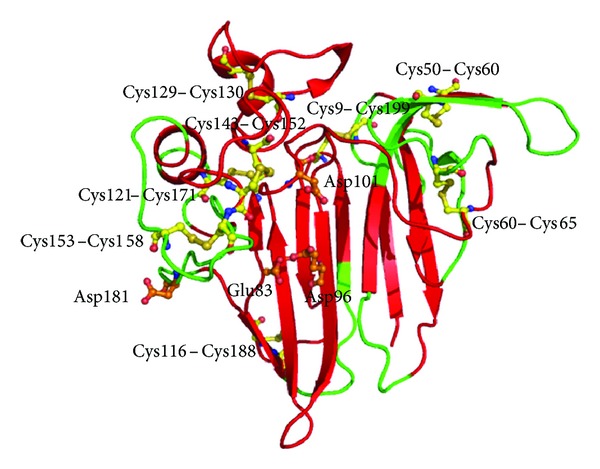
Three-dimensional structure of Banana TLP. Eight disulphide bridges and the acidic residues, Glu 83, Asp96, Asp101, and Asp111 are shown. Twelve amino acid stretches are marked in green.

**Figure 9:**
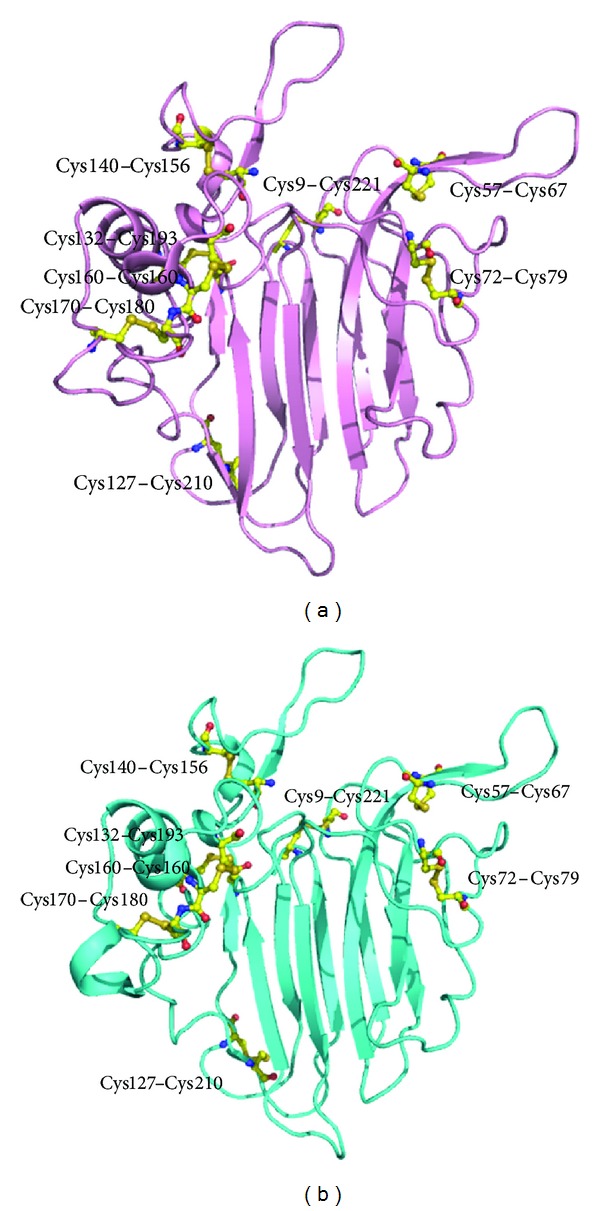
Three-dimensional structures of (a) Pru av 2 and (b) Mal d 2 showing the conserved cysteine residues.

The crystal structure of allergenic TLP from banana (Mus a 4) has been characterized [132]. The protein has three distinct domains: the core domain constituted by a *β*-sandwich formed by two *β*-sheets, an extended *α*-helix domain, and a third domain with a hair-pin segment of two short *β*-strands connected to an extended loop. The structure is stabilized by eight disulphide bridges (Figure 8) which are conserved in other thaumatin-like molecules. A central cleft comprising acidic residues Glu83, Asp96, Asp101, and Asp181 imparts a strong electronegative character. Twelve highly exposed flexible linear epitopes for IgE binding have been speculated in banana TLP. Pru av 2 (PDB: 2AHN) and Mal d 2 (PDB: 3ZS3) have similar overall three-dimensional structure, conserved cysteine residues (Figure 9), and share about 72.9% sequence identity with each other.

Osmotin has been used in production of transgenic crops because of its ability in permeabilizing the plasma membrane and dissipating the membrane pH gradient of the fungal species [133]. However, osmotin was identified as a potential allergen and showed cross-reactivity with allergens from tomato and apple [134]. Three possible antibody recognition sites have been speculated in osmotin and validated by *in vitro* experiments [135].

### 3.6. PR-8 Family Allergens

PR-8 proteins comprise chitinases belonging to class III type having lysozyme activity [136]. One of the major latex proteins, representing this group, is hevamine (Hev b 14) which displays both lysozyme and chitinase activity. It is a 30 kDa basic chitinase from lutoid bodies of the latex of *Hevea brasiliensis* belonging to the family 18 glycosyl hydrolases and has been identified as an allergen present in latex products [137]. Hevamine plays an important role in the self-defense of the rubber tree against pathogenic fungi. However, unlike lysozyme, hevamine cleaves peptidoglycan between the C-1 of N-acetyl glucosamine and the C-4 of N-acetylmuramate.

**Figure 10:**
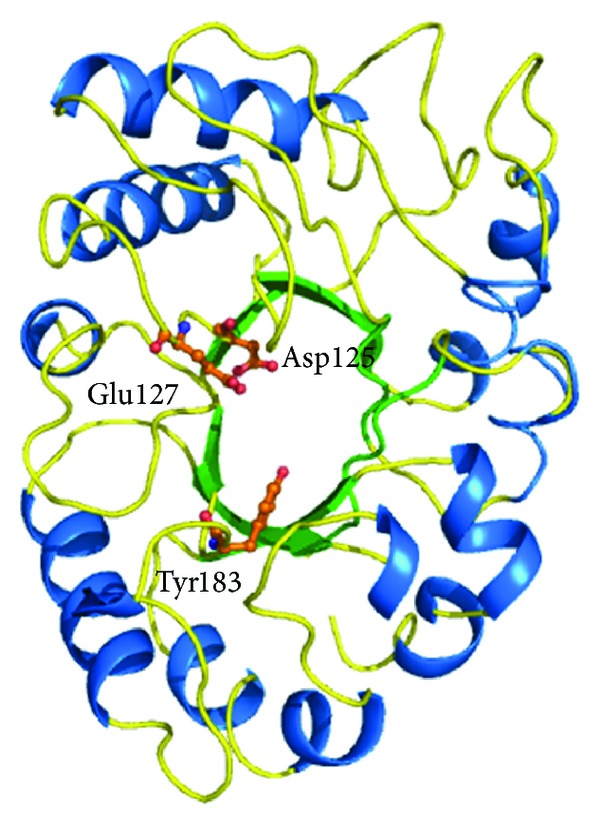
Overall structure of hevamine showing TIM barrel domain and the catalytic residues (in orange).

The amino acid sequence of hevamine shows significant similarity to those of other chitinases/lysozymes from plants and fungi, while there is a lower similarity to chitinases from bacteria, insects, and viruses. The structure of hevamine has a single TIM-barrel (*βα*)_8_ fold with active site residues Asp125, Glu127, and Tyr183 and represents a new class of polysaccharide-hydrolyzing enzymes (Figure 10). The substrate specificity of this protein resides in the loops following the barrel strands that form the substrate binding site. The protein has the two family 18 consensus regions roughly corresponding to the third and fourth barrel strands [138]. Hevamine has been found to be one of the major latex allergens having IgE binding characteristics prevalent mainly in the health care workers of Taiwan [139, 140]. However, the details of antigenic determinants and their role in allergenicity are still unknown.

A similar class III chitinase, Ziz m 1, had been identified in Indian jujube (*Ziziphus mauritiana*) as one of the major allergens having IgE cross-reactivity with the latex allergen [141]. Two stretches of residues from Asn72 - Glu86 and from Val292 - Pro320 are the possible IgE binding epitopes characterized in Ziz m 1 [142]. Recent reports suggest that this chitinase can stimulate multiple cytokines, mainly interleukin-13, from peripheral blood mononuclear cells of latex fruit allergic patients [143]. Another chitinase III protein, Cof a 1 having allergenic potential, has been identified in coffee (*Coffea arabica*) [144].

### 3.7. PR-10 Family Allergens

PR-10 family proteins are intracellular proteins with unknown enzymatic function. Some proteins of PR-10 family are induced under various stress conditions and act as common allergens [145, 146]. However, few PR-10 proteins are also constitutively expressed, indicating a role of these proteins in plant development [147]. The members of this family have low molecular weight (around 15-16 kDa) and are slightly acidic, resistant to proteases, and mostly intracellular and cytosolic [148, 149]. PR-10 proteins are structurally not related to any other class of PR proteins. Apart from direct function in defense, these proteins are implicated in a general function during overall stress as well as during physiological changes in certain developmental stages [150].

Though this family of proteins is widely studied, the exact function of these proteins is still unclear. Some of these proteins are suggested to have a protective role because they are induced when plants undergo pathogenic or environmental stresses. However, some members are also constitutively expressed indicating a general biological role in plant development associated with these proteins. PR-10 proteins are encoded by multigene families which accounts for their multifunctional behavior [151]. Thus PR-10 proteins are not responsible for a particular function in the plant system but some of their conserved sequences and their wide spread existence suggest a crucial role of these proteins [152].

This class of proteins was first identified from parsley [153] followed by other common allergens found in birch pollen [154], celery [155], apple [156], and other fruits and vegetables. Of the different PR-10 proteins, birch-related pollen allergens are extensively studied. The 17.5 kDa protein Bet v 1 is mostly responsible for birch pollen allergy and patients allergenic to birch pollens have been found to develop specific IgE towards Bet v 1 [157]. Thirteen different isoforms of Bet v 1 were identified with the isoform Bet v 1a exhibiting the highest and Bet v 1l exhibiting the lowest allergenic activity [158].

**Figure 11:**
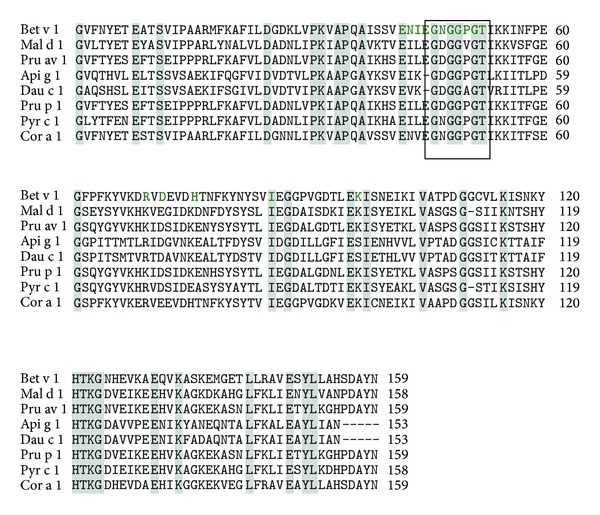
Multiple sequence alignment of birch pollen allergen Bet v 1 with food allergens: Mal d 1, Pru av 1, Api g 1, Dau c 1, Pru p 1, Pyr c 1, and Cor a 1. The identical sequences are highlighted in grey. The residues of Bet v 1 responsible for IgE binding are marked in green. The nearly conserved glycine rich loop is highlighted in box.

Cross-reactivity between Bet v 1 and other food allergens leads to clinical oral allergy syndrome. Several of such allergens homologous to Bet v 1 have been characterized from apple (Mal d1) [156], sweet cherry (Pru av 1) [159], celery (Api g 1) [155], carrot (Dau c 1) [160], peach (Pru p 1) [161], and pear (Pyr c 1) [162]. A sequence comparison of some of these homologous allergens has been done showing a significant sequence similarity of Bet v 1 with the food allergens: about 52.5% with Mal d 1, 57.8% with Pru av 1, 39.8% with Api g 1, 35.9% with Dau c 1, and about 55.0% and 56.6% with Pru p 1 and Pyr c 1, respectively (Figure 11). Cross-reactivity is common when the IgE antibodies, produced originally in response to Bet v1 sensitization, recognize similar epitopes present on the surface of these food allergenic proteins [163]. PR-10 proteins responsible for allergenic reactions to legumes are also homologous to Bet v 1 and have been reported from soy (Gly m 4) [164], peanut (Ara h 8) [165], and mungbean seedlings (Vig r 1) [166]. Birch pollen allergenicity has been also associated with allergenicity from hazelnuts and chestnuts. Cor a 1 [167] and Cas s 1 [168] are the major allergens responsible for allergenicity from hazelnut and chestnut, respectively. Bet v 1 shares maximum sequence similarity (80.5%) with Cor a 1 (Figure 11).

**Figure 12:**
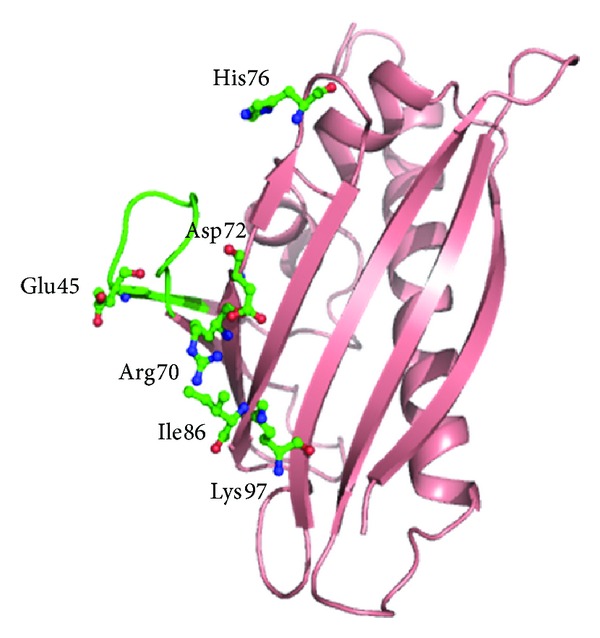
Overall structure of Bet v 1 protein showing the conformational epitope formed by amino acid residues from Glu42 to Thr52 (in green) and additional dispersed amino acids Arg70, Asp72, His76, Ile86, and Lys97 (in green) for Fab binding. The critical residue for antibody binding Glu45 is marked.

Bet v1 consists of seven stranded *β*-sheet structure wrapped around a 25-residue long C-terminal *α*-helix structure both of which are separated by two consecutive helices (Figure 12). A hydrophobic cavity is created by the hydrophobic residues clustering in the interior region with some polar residues pointing into the core [169]. The presence of such an internal cavity suggests a possible role in binding with hydrophobic ligands. A glycine rich loop connecting the 2 *β*-strands, *β*2 and *β*3 is nearly conserved among all the homologous PR-10 proteins. This might have a putative role in lipid binding [170].

Some of the ligands binding to Bet v 1 with low micromolar affinity such as fatty acids, flavonoids, and cytokinins have been identified. The crystal structure of hypoallergenic isoform Bet v 1l in complex with deoxycholate suggests that Bet v 1 homologous proteins can act as a general plant steroid carrier [171]. It has been evaluated from the crystal structure of this birch pollen allergen in complex with the Fab fragment of a murine monoclonal IgG antibody (BV16), that the centrally located residue Glu45 is critical for antibody recognition and forms two hydrogen bonds with the heavy-chain variable region of the Fab fragment [172]. A conformational epitope containing amino acid residues from Glu42 to Thr52 (which also contains the glycine rich loop) and additional dispersed amino acids Arg70, Asp72, His76, Ile86, and Lys97 has been identified to be responsible for binding to antibody (Figure 12).

The secondary structure and the tertiary fold of Pru av 1, Api g 1, and Dau c 1 are identical to Bet v1 [173]. They have a large internal hydrophobic cavity that can interact with hydrophobic ligands and are important for physiological functions. The hydrophobic cavity is large enough to accommodate two such molecules. Binding studies of various phytosteroids to Pru av 1 suggest similar interactions like Bet v 1. Since both of these proteins have high level of sequence identity and similar overall backbone conformation, they share identical molecular surface in terms of shape and charge distribution. This explains the prevalence of cross-reactive IgE binding epitopes in these proteins [174]. The epitope for antibody binding in Bet v 1 has been found to be nearly conserved in Pru av 1 with Glu45 which may play a similar role in this molecule.

**Figure 13:**
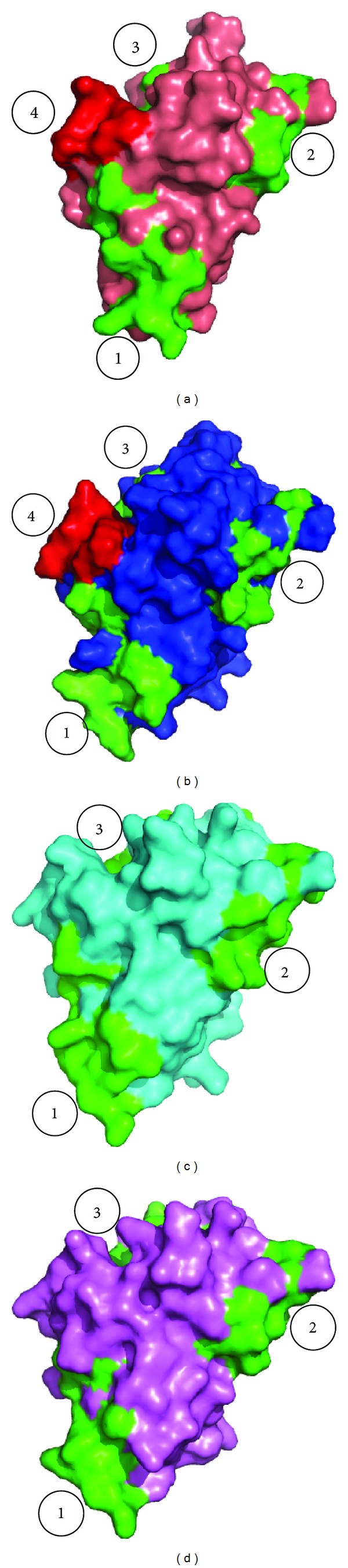
The three common surface epitopes (in green) recognized for IgE binding marked from 1 to 3 in (a) Bet v 1, (b) Pru av 1, (c) Api g 1, and (d) Dau c 1. The conformational epitope required for Fab binding (in red) in Bet v 1 (marked 4) is nearly conserved in Pru av 1 but absent in Api g 1 and Dau c 1 because of the absence of Glu45.

Some structural differences exist between Bet v 1 with Api g 1 and Dau c 1 although these allergens exhibit cross-reactivity among themselves [160, 175]. The latter two share about more than 80% sequence similarity with each other. Api g 1 lacks the negatively charged Glu45, in contrast to Bet v 1 and Pru av 1 [175]. The epitopes responsible for binding are found to be different from those elucidated by the structure of Bet v 1 in complex with Fab fragment of monoclonal BV16 antibody. Three conserved surface patches responsible for IgE binding are found to be common among Bet v 1, Pru av1, Api g 1, and Dau c 1 (Figure 13). It has also been observed that cross-reactivity between Bet v 1 and Mal d 1 occurs not only at the serologic level but also at the level of allergen specific T helper cells [176]. Eight cross-reacting T cell epitopes have been observed between the two allergens.

### 3.8. PR-14 Family Allergens

PR-14 proteins are identified as lipid transfer proteins (LTPs) originally named after their ability to transfer phospholipids and other fatty acid groups across cell membranes. They are highly conserved group of small proteins with molecular weights in the range of 9-10 kDa present in high amounts in higher plants and can also bind to acyl groups. These proteins are present in significant amounts in vascular tissue and in the outer cell layers of plants. They are involved in plant defense against bacterial and fungal pathogeneses as well as under different environmental stresses such as drought, heat, cold, or salt [177, 178]. There are evidences which suggest that LTPs are also involved in cutin formation, where they act as carriers of acyl monomers and in the process of cell wall extension [179]. They are divided into two types: those specific for certain classes of phospholipids and those that are able to accommodate several lipid classes, called nonspecific LTPs. Allergenic features of nonspecific LTPs (ns-LTPs) were reported in fruits, vegetables, nuts, pollen, and latex [180]. Due to their extreme proteolytic resistance, these allergens are able to traverse up to the gastrointestinal immune system, allowing sensitization and inducing specific IgE thereby eliciting severe clinical symptoms [181].

**Figure 14:**
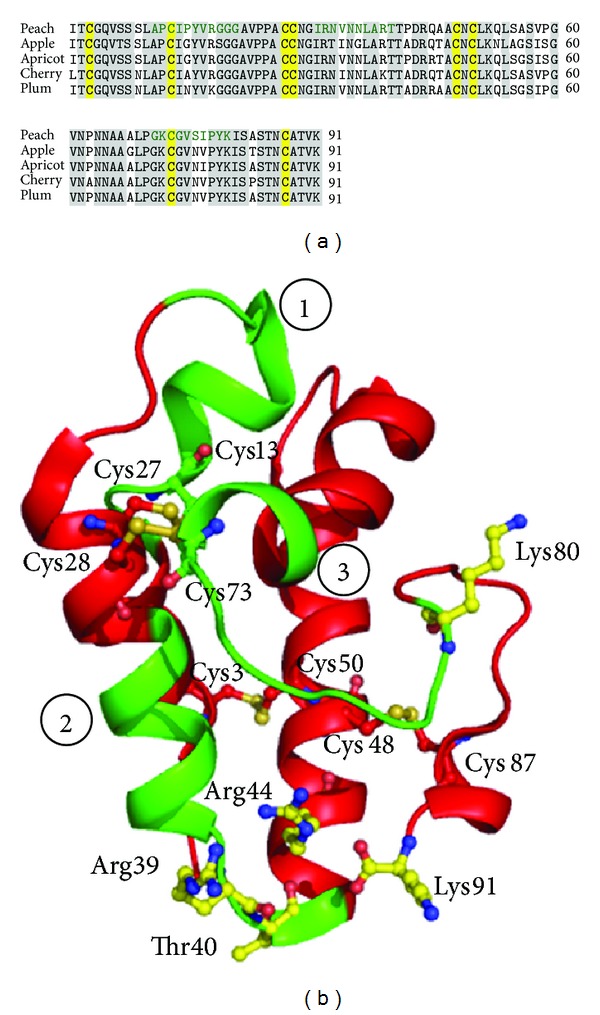
(a) Multiple sequence alignment of allergens of ns-LTPs from four Rosaceaefruits: peach, apple, apricot, and cherry. The identical sequences are highlighted in grey. Eight conserved cysteine residues are highlighted in yellow. The speculated three IgE binding epitopes of Pru p 3 from peach are marked in green. (b) Overall structure of ns-LTP, Pru p 3 showing the three possible IgE epitope binding regions (in green) marked from 1 to 3. The five charged residues in yellow having a possible role in epitope recognition and the eight cysteine residues forming four disulphide bridges are also marked.

LTPs are the most important allergens of the Rosaceaefruits, such as peach (Pru p 3) [17], apple (Mal d 3) [182], apricot (Pru ar 3) [183], cherry (Pru av 3) [184], and plum (Pru d 3) [185], when no pollinosis is involved. Due to significant sequence identity (more than 81%) (Figure 14(a)) shared by ns-LTPs from Rosaceae fruits along with the considerable degree of immunological cross-reactivity, it has been suggested that they have comparable IgE binding epitopes [186]. Patients allergic to PR-14 proteins in fruits tend to have a higher rate of anaphylaxis (36%) than patients having fruit allergy by PR-10 proteins (18%).

The structure of the allergen belonging to this family, Pru p 3 from peach, has been extensively studied [187]. The main structural motif is represented by an *α*-helical compact domain, where four helices are connected by short loops (Figure 14(b)). Eight conserved cysteine residues were observed forming four disulfide bridges which makes it highly resistant to harsh temperature and pH changes. It had been speculated that five positively charged residues, Arg39, Thr40, Arg44, Lys80, and Lys91, are the possible candidates involved in epitope formation. Moreover, using a library of 10-mer synthetic peptides, which screened the whole protein sequence, three potential IgE-binding epitope regions have been identified [188] (Figure 14(b)), which are nearly conserved in other LTPs of Rosaceaefruits (Figure 14(a)).

Recent reports have also indicated that ns-LTPs from species other than Rosaceae, such as nuts, cereals, grapes, oranges, and vegetables, might also be involved for plant food allergies [189]. Severe reactions against hazelnut and chestnut are linked to sensitization to the LTPs, Cor a 8 [190], and Cas s 8 [191], respectively. The major allergens of maize (Zea m 14) [192] and barley (Hor v 14) [193] are also reported to be LTPs and are highly homologous with the peach LTP. LTPs of PR-14 family are also reported from pollens including Par j 1 from weeds of *Parietaria judaica* affecting 50% of allergic patients in the Mediterranean area [194].

## 4. PR-Proteins and Disease Resistant Genetically Modified Crops 

PR proteins are of immense importance as preservative agents in food industry and for producing disease resistant plants by genetic engineering [195]. Various studies have revealed that transgenic plants overexpressing genes of the PR-1, PR-2, PR-3, and PR-5 families mediate host plant resistance to phytopathogenic fungi [196–198]. Coexpression of multiple antifungal protein genes in transgenic plants seems to be more effective than expression of single genes [199]. It is possible that such genetically modified (GM) plants with enhanced expression of PR proteins will also be associated with increased allergenicity and toxicity thus raising a serious question for their commercial acceptability. PR protein, osmotin used for developing transgenic crops, showed cross-reactivity with tomato and apple allergens [134].

Different strategies are adopted to monitor the transformed crops for their allergenicity and various guidelines are defined by the Food and Agriculture Organization (FAO), World Health Organization (WHO), and Codex Alimentarius Commission (Codex) to determine whether a new GM crop can be commercialized [200]. The present approach uses a combination of methodologies to evaluate the allergenicity assessment [201]. The allergenicity potential of modified food is primarily speculated on the basis of whether the source of the transformed protein is a plant known to produce allergen and also on the characteristic features of the introduced protein. The homology of the latter to known allergens along with IgE reactivity of the transformed protein with individuals with known allergies to the original source of the novel gene or related allergies is assessed. Moreover, the resistance of the novel protein to pepsin and the immunoreactivity of the novel protein in appropriate animal models are also evaluated [202]. Such step by step approach will provide valuable insights to estimate whether the transformed protein will be allergenic or not.

## 5. Conclusions

PR proteins and their homologues are responsible for the defense against various stresses including pathogen attacks, wounding, use of chemicals, and pollutants. Recent studies have suggested their immense importance in agricultural and food industry with the introduction of transgenic plants. However, many of these protective proteins of the plants have demonstrated allergenicity specially the PR proteins belonging to the families 1, 2, 3, 4, 5, 8, 10, and 14. Though there are not much similarities among the different families of PR proteins in terms of their sequence identities or structures, similarity in the amino acid sequences among allergens from diverse plants within the same family results in cross-reactivity. The allergenicity of the PR proteins is also guided by several environmental factors like the use of chemical inducers in agriculture and environmental pollutants. Exploring new PR proteins implicated in allergenicity and a complete understanding of their structures and IgE binding epitopes are necessary for their safe use in plant engineering. The knowledge of the localization of IgE epitopes on the allergen helps in the identification of cross-reactivity among homologous proteins and may also contribute to the design of effective immunotherapy strategies for certain allergy producing substances like latex, pollen, and so forth along with their respective related allergies.

## Figures and Tables

**Table 1:** Different PR-protein families and allergens identified.

Family	Proteins	Functions	Allergens identified with source and allergenic symptoms
PR-1	PR-1 a, PR-1 b, and PR-1 c	Antifungal	Cuc m 3 (muskmelon)—oral allergy syndrome
PR-2	*β*-1,3-Glucanases	Cleaves *β*-1,3-glucans	Hev b 2 (latex)—contact dermatitis Ole e 9 (olive)—respiratory allergy Mus a 5 (banana)—oral allergy syndrome
PR-3	Chitinase types I, II, IV, V, VI, and VII	Endochitinase	Pers a 1 (avocado)—itchy eyes or nose, asthma, swelling, and so forth. Mus a 2 (banana)—food allergy like swelling of lips, anaphylaxis, and so forth
PR-4	Chitinase types I and II	Antifungal and chitinase	Hev b 6.01, Hev b 6.02, and Hev b 6.03 (latex)—contact dermatitis
PR-5	Thaumatin-like proteins	Antifungal	Jun a 3 (mountain cedar), Cry j 1 (Japanese cedar), and Cup a 3 (Arizona cypress)—rhinitis, conjunctivitis, and asthma Pru av 2 (cherry), Mal d 2 (apple), Cap a 1 (bell pepper), Act d 2 (kiwi), and Mus a 4 (banana)—oral allergy syndrome
PR-6	Tomato proteinase inhibitor I	Proteinase inhibitor	—
PR-7	Tomato endoproteinase P	Endoproteinase	—
PR-8	Cucumber chitinase	Chitinase III	Hevamine (latex)—contact dermatitis. Ziz m 1 (Indian jujube)—oral allergy syndrome Cof a 1 (coffee)—eye and airway allergy
PR-9	Tobacco lignin-forming peroxidase	Peroxidase	—
PR-10	Parsley “PR-1” Bet v 1, Mal d 1, Api g 1, and Dau c 1	Ribonuclease-like	Bet v 1 (birch pollen)— allergic rhinoconjunctivitis and asthma Pru av 1 (cherry), Mal d 1 (apple), Api g 1 (celery), and Dau c 1 (carrot)—oral allergy syndrome Gly m 4 (soy), Vig r 1 (mung bean), Cor a 1 (hazelnut), and Cas s 1 (chestnut)—oral allergy syndrome
PR-11	Tobacco chitinase type V	Chitinase	—
PR-12	Radish Rs-AFP3	Defensin	—
PR-13	*Arabidopsis* THI2.1	Thionin	—
PR-14	Lipid transfer proteins	Shuttling of phospholipids and fatty acids	Par j 1 (weed)—rhinitis and asthma Pru p 3 (peach), Mal d 3 (apple), Pru av 3 (cherry), Pru ar 3 (apricot), Cor a 8 (hazelnut), Cas s 8 (chestnut), and Zea m 14 (maize)—oral allergy syndrome
PR-15	Barley OxOa	Oxalate oxidase	—
PR-16	Barley OxOLP	Oxalate-like oxidase	—
PR-17	Tobacco PRp27	Unknown	—
